# ‘Doing the Right Things’ First and Then ‘Doing Things Right’

**DOI:** 10.1111/jep.70079

**Published:** 2025-04-01

**Authors:** James A. Marcum

**Affiliations:** ^1^ Department of Philosophy Baylor University Waco Texas USA

Modern medicine is facing a crisis with respect to providing the quality of healthcare patients seek or need, especially for chronic diseases such as cancer and diabetes. Sturmberg and Mercuri offer an insightful solution to this crisis with a shift in perspective. Invoking the management guru, Peter Drucker [1], they argue that the necessary shift in perspective is from ‘Doing things right’ to ‘Doing the right things’ first. In other words, medicine needs to shift to a perspective that includes the context in which the patient is embedded, from one that often ignores or excludes the patient's context and thereby fails to deliver the healthcare that the patient really requires. The shift not only has an impact on medical research but also on clinical practice. Indeed, the patient's lived context or lifeworld is important with respect to providing the healthcare patients seek or need.

Sturmberg and Mercuri also invoke the mathematician, David Sumpter [2], and his four ways of thinking to implement the paradigmatic shift from ‘Doing things right’ to ‘Doing the right things’ first. According to Sumpter, the first way of thinking is statistical by which the individual patient is defined in terms of the collective average. The next is interactive thinking, which explicates collective interactions simply with respect to a single linear cause and effect. The third is chaotic thinking in which the randomness and disorder of events are factored into the cognitive equations and calculations. The final is complex thinking, which, according to Sturmberg and Mercuri, is ‘more focused on introspection and self‐reflection…about finding the stories which help us to better understand ourselves, as well as those around us’ (p. 9). Their goal for contemporary medicine, then, is to shift from statistical and interactive thinking that only considers the average and a linear cause and effect in terms of ‘Doing things right’ to complex and chaotic thinking that embeds the components within a multilayered and multifactorial context or story including the ethical with respect to ‘Doing the right things’ first.

Besides Sumpter, Sturmberg and colleagues [3, 4] have also drawn on another management guru, David Snowden, and the Cynefin framework to address multilayered and multifactorial contexts [5, 6], which is relevant to Sturmberg and Mercuri's current project. The Cynefin framework consists of four domains for making decisions: clear, complicated, complex and chaotic. The clear domain pertains to knowing the relevant factors involved in a decision, while the complicated domain involves several unknown factors but still a robust decision is possible. Both complex and chaotic domains contain a majority of unknown factors or unknowable factors, respectively, such that decisions are often difficult and questionable but still need to be made. The relevance for the Cynefin framework for Sturmberg and Mercuri's project is that chronic diseases inhabit complex and chaotic domains and that both clinical research and practice require discovering the interdependent causes and effects associated with such diseases. To that end, the shift requires a change in fundamental philosophical assumptions.

As Sturmberg and Mercuri note, the standard approach to biomedical research and its clinical practice is reductionistic. And in terms of Snowden, complex and chaotic systems are unfortunately often reduced to clear and simple ones inappropriately. The standard biomedical approach, then, generally involves ‘Doing things right’ initially with respect to solving the problem correctly in terms of pathological mechanisms of chronic diseases. The result of this approach is that the patient is often fragmented into individual body parts to identify which part is broken. Thus, there is a 'right' way to fix the part, in contrast to a ‘wrong’ way. And in terms of Drucker, statistical and interactive thinking as reductive generally rely on a linear relationship between a single cause and effect in ‘Doing things right.’ According to Sturmberg and Mercuri, the term ‘right’ is used from an epistemic perspective—knowing how to do things right technically. But as they argue, the term ‘right’ can also be used ethically. In other words, ‘Doing the right things’ first involves engaging primarily in what is morally right or appropriate under the given circumstances or context, in contrast to what is morally wrong. As Sturmberg and Mercuri stress, this context is often ignored when doing things like conducting medical research or providing clinical care. And as they contend, complex and chaotic phenomena, such as chronic illnesses, must be examined as a system or network of causes and effects to understand the phenomenon wholly. Rather than making the problem simple when indeed it is complex, if not chaotic, complex and chaotic thinking rely on a holistic assumption that maintains and requires the problem's multilayered and multifactorial context.

Mary O'Flaherty Horn [7] provides a good example of why the patient's context is important for delivering quality clinical care. Diagnosed with amyotrophic lateral sclerosis (ALS), Horn traveled almost 2000 miles for a second opinion. Before that opinion, she had to undergo an electromyography. As Horn narrates the story, the clinician, Dr. L., certainly was able to perform the electromyography competently or ‘Doing things right.’ What the clinician was unable to do was to conduct the procedure humanely or ‘Doing the right things.’ And so Dr. L. was capable of ‘Doing things right,’ that is preforming the electromyography to determine the extent of Horn's ALS, but incapable of ‘Doing the right things’ first, that is conducting the procedure with the realisation of her vulnerable condition physically, emotionally, and existentially. As Horn concludes her humiliating experience with the clinician, ‘Encounters such as mine with Dr. L., the antithesis of caring, could become more common as medical care becomes more fragmented and long‐term relationships with patients become relics’ ([7], p. 940). Indeed, medicine with its emphasis on ‘Doing things right’ first has placed the cart before the horse. And Sturmberg and Mercuri's clarion call is to place the horse in its correct position both in terms of medical research and clinical practice.

Finally, Sturmberg and Mercuri begin their article with two epigraphs, the first by Einstein, ‘It is the theory which decides what can be observed,’ and the other by Planck, ‘If you change the way you look at things, things you look at change’ (p. 2). Observations are certainly theory‐laden [8]; but Einstein's quote, as Sturmberg and Mercuri argue, ‘overlooks our ability to critically analyse our observations to form theory’ (p. 3). In other words, theories may also be observation‐laden [9], which leads to ‘Planck's insight that changing the way we look at things will alter our understandings about things’ (p. 2). In Kuhnian terms, an ‘essential tension’ exists between the theory‐ladenness of observations and the observation‐ladenness of theories or between ‘the traditionalist and the iconoclast’ scientist ([10], p. 227). In other words, the normal scientist works within a traditional or paradigmatic framework in which theories or paradigms drive observations, while the revolutionary scientist works within an iconoclastic framework in which (anomalous) observations drive theories in terms of a paradigmatic shift [11]. And this shift can result in significant world changes [12]. In sum, the paradigmatic shift Sturmberg and Mercuri are boldly advocating is a change in the medical world from a simple biomedical model to a systems‐network model that avoids ‘Doing harm’ by ‘Doing good’ with respect to ‘Doing the right things’ first and then ‘Doing things right.’

## Conflicts of Interest

The author declares no conflicts of interest.

## Data Availability

The author has nothing to report.

## References

[1] P. F. Drucker , The Essential Drucker: The Best of Sixty Years of Peter Drucker's Essential Writings on Management (Taylor & Francis, 2001).

[2] D. Sumpter , Four Ways of Thinking: Statistical, Interactive, Chaotic, and Complex (Penguin Books, 2023).

[3] J. P. Sturmberg and J. A. Marcum , “From Cause and Effect to Causes and Effects,” Journal of Evaluation in Clinical Practice 30, no. 2 (2024): 296–308, 10.1111/jep.13814. 36779244

[4] J. P. Sturmberg and C. M. Martin , “COVID‐19—How a Pandemic Reveals That Everything Is Connected to Everything Else,” Journal of Evaluation in Clinical Practice 26, no. 5 (2020): 1361–1367, 10.1111/jep.13419. 32633056 PMC7362160

[5] C. F. Kurtz and D. J. Snowden , “The New Dynamics of Strategy: Sense‐Making in a Complex and Complicated World,” IBM Systems Journal 42, no. 3 (2003): 462–483, 10.1147/sj.423.0462.

[6] D. Snowden and A. Rancati , Managing Complexity (and Chaos) in Times of Crisis: A Field Guide for Decision Makers Inspired by the Cynefin Framework (Publications Office of the European Union, 2021).

[7] M. O. Horn , “The Other Side of the Bed Rail,” Annals of Internal Medicine 130, no. 11 (1999): 940–941, 10.7326/0003-4819-130-11-199906010-00020. 10375344

[8] N. Hanson , Patterns of Discovery: Inquiry Into the Conceptual Foundations of Science. (Cambridge University Press, 1958).

[9] I. Votsis , The Observation‐Ladenness of Theory, https://www.votsis.org/PDF/Votsis_Abstract_Observation_Ladenness.pdf.

[10] T. S. Kuhn , The Essential Tension: Selected Studies in Scientific Tradition and Change (University of Chicago Press, 1977).

[11] T. S. Kuhn , The Structure of Scientific Revolutions, 2nd ed. (University of Chicago Press, 1970).

[12] P. Horwich , ed., World Changes: Thomas Kuhn and the Nature of Science. (MIT Press, 1993).

